# Good Management Practice Is Correlated With Good Performance of Community-Engaged Primary Health Care Facilities in Peru

**DOI:** 10.9745/GHSP-D-23-00402

**Published:** 2024-08-27

**Authors:** Laura C. Altobelli

**Affiliations:** a Future Generations University, Franklin, WV, USA.; b School of Public Health and Administration, Cayetano Heredia University, Lima, Peru.

## Abstract

**Background:**

Increasing prioritization of quality primary health care (PHC) includes community engagement as a key element to improve PHC performance. We assessed the correlation of good regional management practices with PHC performance in Peru in community-comanaged PHC that is designed with multiple accountability mechanisms.

**Methods:**

We conducted a secondary analysis of a survey of Dirección Regional de Salud (regional health directorates, DIRESAs) regarding their management of public PHC services with collaborative community involvement by a Comunidad Local de Administración de Salud (Local Community for Health Administration, CLAS). CLAS-run facilities have previously shown evidence of superior performance over standard PHC services. We classified survey questions on 5 management functions of the Primary Health Care Performance Initiative: leadership, information system, financial control, transfer of management and leadership skills to health facilities, and supervision. An expert panel designated management practices as “good” or “less effective.” The outcome, PHC service performance, was the percentage of CLAS comanaged facilities in each DIRESA achieving coverage and utilization goals. We correlated frequency of good management practices with PHC service performance. DIRESAs were divided into Group 1, “higher performance,” and Group 2, “lower performance,” to identify specific practices linked to better performance.

**Results:**

We identified 32 good management practices among 52 response options to 17 questions. Correlation between good management practice and good service performance was significant (r=.7266; 12 df; *P*<.01). An average of 91.1% and 37.6% of CLAS facilities achieved service goals in Groups 1 and 2, respectively. Of all good management practices identified, an average of 40.6% and 24.0% were used by Groups 1 and 2, respectively. Group 1 used 11 specific good practices more frequently than Group 2.

**Conclusions:**

Regional management and community-comanaged PHC services designed with accountability mechanisms should be intentionally aligned, incorporating these into policies, budgets, processes, and capacities to strengthen PHC services.

## Abstract (precis)

This study aims to contribute to a better understanding of regional management practices that could support primary health care service performance in the context of the innovative community engagement model implemented through the unique program with embedded mechanisms for accountability.

## Teaser

**Key Findings:**

- In a survey of regional health directorates regarding their support to primary health care (PHC) services comanaged by community nonprofit organizations, good regional management practices were significantly correlated with improved PHC service performance.
- Specific management practices that were linked to better performance of comanaged PHC services included: formal appointment of regional management team leadership, vigorous financial oversight (periodic site visits for document review and external audits), provision of training and information to the community nonprofit committee and PHC staff, supervision visits by program specialists, and inclusion of health promotion in local health plans.

**Key Implications:**

- Policymakers should intentionally design regional management structure and roles that are aligned with and hold accountable PHC services with community-comanaged PHC.
- Policymakers need to carefully design and support the structure, roles, and institutionalization of community-comanaged PHC facilities to include legally enforced accountability mechanisms for human resources and local purchasing that have been shown to improve quality, equity, and coverage of PHC services.
- Health sector officials and functionaries need to oversee implementation and continuous evaluation of regional management practices that support and hold accountable redesigned PHC with community roles for collaborative management.

## INTRODUCTION

Primary health care (PHC) is credited as the main vehicle for achieving universal health coverage (UHC) and the Sustainable Development Goals.^1^^–^^3^ The Alma Ata Declaration on Primary Health Care of 1978 was signed by 134 countries^4^; 120 of them reconfirmed their commitment with the 2018 Declaration of Astana.^5^ These have generated numerous global and national policies and calls to action for UHC through PHC.^6^^–^^11^ In 2024, the World Health Organization (WHO) Alliance for Health Policy and Systems Research identified 5 focus areas where health policy and systems research can make a unique contribution to improving health and health equity, the first being the reform of health systems and services to achieve UHC through a PHC approach.^12^

The urgency of equitable health systems reform was made apparent in the major failings of PHC in many low- and middle-income countries during the COVID-19 pandemic: 4.5 billion people were not covered by essential health services in 2021 and “less than a third of countries have improved health service coverage” over the past 2 decades, with disproportionate consequences for the poor.^3^

The 2020 WHO operational framework for PHC provided 4 strategic and 10 operational levers to guide the substantial reforms that are required to meet UHC goals, the 4 main levers being political commitment and leadership, governance and policy, funding and allocation of resources, and engagement of communities and other stakeholders.^2^ This and other conceptual frameworks, such as the Primary Health Care Performance Initiative^6^ and Essential Public Health Functions,^13^^,^^14^ help countries identify what works for better delivery of quality population-based PHC services and where more innovation and research are needed.

Governance and leadership are critical strategic levers for PHC but have had relatively little research published on them compared to other domains.^15^^,^^16^ Although governance and management comprised 1 of WHO’s 6 “Building Blocks,” there was little consensus on key concepts and measures of it.^17^ Definitions of PHC governance now include quality management infrastructure that has varied aspects and several operational levels; it can refer to the way services are managed in health facilities or at higher levels, such as district or subnational management units.^6^ PHC systems with optimal management practices are part of the structural innovations that are “more likely to improve service quality substantially and at scale than are micro-level efforts.”^18^

Governance and leadership are critical strategic levers for PHC but have had relatively little research published on them compared to other domains.

In general, management practices have been categorized as leadership, planning, monitoring, supervision, and evaluation.^19^ Evaluation detects differences between the actual and desired situation based on planning. Monitoring and supervision are mechanisms to provide information for evaluation and subsequent replanning.

The Primary Health Care Performance Initiative suggested roles for PHC management as qualified management and leadership, information systems to support and improve quality, budgets and financing systems to meet essential costs, management capability and leadership, and supportive supervision for continual improvement of knowledge and skills.^6^

The WHO PHC operational framework proposes a set of governance roles at the subnational level (Box 1).^2^

BOX 1 Subnational-Level Governance Roles in the World Health Organization Operational Framework for Primary Health Care

- Reform and align the integrated primary health care (PHC)-oriented governance mechanism and planning processes at the subnational level to respond to its 3 components (integrated health services including public health functions, community participation, and multisectoral collaboration).
- Create community-based multistakeholder forums for collective accountability and action on health and health-related issues.
- Create an organizational culture that supports monitoring and evaluation through knowledge-sharing, open feedback, and a demand for data in decision-making processes.
- Strengthen PHC-oriented management protocols that encourage provider report cards, patient satisfaction surveys, patient-reported outcomes, and balanced scorecards.
- Support public, private, and community actors to develop competencies for engaging across the PHC components.

Source: World Health Organization.^2^

Good practices for management of health policy implementation cannot be generalized and need to be tailored to each country.^20^ When policies lack clarity on management responsibilities, scant priority is assigned for effective control of PHC from national and subnational levels. Recent PHC norms and legislation in Peru may be useful technical models and documents but have little content on mechanisms and capacities for subnational management structure and leadership that are needed to support health facility-level management and services.^21^^–^^24^ Traditional public PHC services in Peru are managed from the top down for all goods and services. Local health facilities have no responsibility for planning, financial, or human resource management and have no accountability or community engagement mechanisms. The focus of supervisory action is on technical protocols and information reporting that is exclusively health service related.

In a study of Peruvian government PHC services, health facility medical officers were asked to identify the most common PHC management problems.^25^ Their responses reflected the most generally identified PHC service delivery bottlenecks, principally focused on human resource shortages, lack of supplies and medicines, poor infrastructure, and budget deficits but not on problems of management per se. A perception of responsibilities in a multilevel working system of clear roles, accountabilities, and principal-agent relationships was lacking in the responses provided.

Community engagement is another domain that has again been brought to the fore by WHO and firmly established as a strategic lever in the operational framework for PHC—both as part of local governance in terms of community voice and oversight for greater social accountability and as a component of population health management, reaching outside the health facility for proactive community-based education and monitoring.^2^^,^^14^

A systematic review of 111 studies was conducted in 2019 on what works and does not work to strengthen PHC systems in low- and middle-income countries. Five areas of intervention were found most likely to be effective for improving the desired outcomes of quality, coverage, efficiency, responsiveness, and equity of PHC service delivery: (1) placing greater emphasis on use of nonphysician health workers, (2) integrating noncommunicable disease prevention and control into the basic package of care, (3) modernizing PHC information systems, (4) building managerial capacity, and (5) institutionalizing community engagement.^26^ Assessment of managerial capacity was found lacking in nearly all the studies reviewed; nevertheless, it was highlighted as “one of the interventions most likely to improve performance outcomes,” being necessary to operationalize all the other facility-level interventions. Furthermore, the evidence review showed that improvement of all desired outcomes of PHC services was strongly associated with community engagement.

The current study uses a systems-thinking approach to understand an important health system reform in Peru that strengthens PHC. Specifically, this study aims to contribute to a better understanding of regional management practices that could support PHC service performance in the context of the innovative community engagement model implemented through the unique national Shared Administration (SA) Program that has various embedded mechanisms for accountability. Established 30 years ago, community involvement in PHC services in Peru was institutionalized in 1994 through the formation of legally recognized registered community-based nonprofit organizations called Comunidad Local de Administración de Salud (Local Community for Health Administration, CLAS) under the SA program.^27^ The program was so popular that by 2002, it had scaled up, based on demand, to encompass nearly one-third of all public PHC facilities in Peru.

The current study uses a systems-thinking approach to understand an important health system reform in Peru that strengthens PHC.

A 2007 survey, unreported until now, of Dirección Regional de Salud (regional health directorates, DIRESAs) provided the opportunity to assess their management practices in relation to CLAS-comanaged PHC facilities at a time when there was relatively strong national support for the SA program. Studies and evaluations before 2007 uniformly showed significantly better results for CLAS facilities in comparison to non-CLAS facilities on health service indicators.^28^^–^^30^ Furthermore, a national law on comanagement and citizen participation in PHC services was passed in 2007 by the Peruvian Congress to provide legal stability to the SA program and CLAS facilities.^31^ However, since that time, opposition from interest groups, faulty decentralization efforts, an apparently competing model of health service organization, and budget cuts contributed to the eroding of Ministry of Health (MOH) support to CLAS comanagement of PHC facilities. More detail is provided in the Discussion section.

We posit that the 2007 survey remains relevant for assessment for retrospective learning on what works. In the post-pandemic era, a renewed realization that PHC should be prioritized provides the impetus for the consideration of CLAS facilities as a solution to reach UHC. Thus, it is relevant to the future expansion of CLAS facilities to assess factors that helped to make them work well. Furthermore, the 2007 survey results could provide guidance to other countries that are starting to design their own models of PHC with community engagement to better reach UHC by 2030.

## COMMUNITY COMANAGEMENT MODEL THROUGH THE SHARED ADMINISTRATION PROGRAM

The most important policy and structural change of the MOH for PHC in Peru in the last 3 decades was the innovative design and institutionalization of a community comanagement model through the SA Program. The program was established in 1994 by Supreme Decree (a legislative instrument just below the level of a law) that legally recognized CLAS. Beginning with 16 PHC facilities collaboratively administered by CLAS in 1994, by 2006, as many as 764 CLAS had been formed, comanaging 2,155 (31%) PHC facilities of a total of about 6,700, benefiting 7 million inhabitants comprising 80% of the poverty population in Peru.^27^ In 2007, the SA program and the comanagement of PHC facilities by CLAS were formalized into national law with regulations to the law approved in 2008.^31^^,^^32^

The comanagement model for PHC has 7 innovations that incorporate accountability mechanisms, none of which are present in traditional public administration of the health sector.

The comanagement model for PHC has 7 innovations that incorporate accountability mechanisms that are not present in traditional health sector public administration.

1. Involvement of community members in PHC management was legalized through the SA Program. A CLAS is inscribed in the Public Registry as a private nonprofit civil association with its membership elected from communities. The membership elects a Board of Directors from within its ranks, which works with the CLAS manager, who is chief medical officer of the PHC facility (or micro-network of facilities) comanaged by a CLAS. The CLAS collaboratively manages public funds transferred to a shared bank account under a contract with the State with strict rules of accountability. The 2007 CLAS law^31^ modified its membership structure to include representatives of several public entities, including the local municipal government (not the mayor), in addition to a majority of elected community representatives.^33^ Non-CLAS facilities have no community involvement in PHC management.
2. CLAS law allows local health facility staff to be hired and fired using standard private-law labor contracts that generally oblige accountability for quality service. Furthermore, the community has a voice in staff selection and oversight. CLAS comanaged facilities also have government payroll employees on staff since, once they gain such status, they can be fired only for extreme wrongdoing. Non-CLAS facilities are staffed mainly by government payroll employees and some short-term personnel contracted by the DIRESA.
3. New financial mechanisms for PHC were created in which funds from public health insurance per capita payments are transferred to the CLAS comanaged bank account to allow rapid and efficient purchasing of drugs, supplies, equipment, and infrastructure under joint legal control of the CLAS board of directors and CLAS manager with monthly financial reports to the DIRESA. Non-CLAS facilities do not manage funds at the local level.
4. CLAS comanaged facilities have local procurement mechanisms for PHC in which purchasing was modernized to allow use of private-sector law by CLAS comanaged facilities, advancing transparency and efficiency in PHC acquisitions. The community has a voice in spending decisions. In contrast, procurement under public administration law is not done locally; purchasing procedures are cumbersome, less transparent, and have no community involvement.
5. Each CLAS and its comanaged PHC facility(ies) develops an annual local health plan with input from community members to incorporate local priorities. The plan serves as a guide to implement, monitor, and evaluate services and community activities. Non-CLAS facilities have no local health plan and respond primarily to goals established by centrally-determined vertical health programs.
6. Each CLAS President signs a tripartite Co-management Agreement with the DIRESA director and the district mayor based on commitments to implement the local health plan. Among other advantages, this legal agreement allows the district government to channel supplemental funds to CLAS facilities for community health and other activities. Non-CLAS facilities cannot sign agreements with other public or private entities.
7. Public financing of an Equipo Técnico de Administración Compartida (Technical Team for Shared Administration, TTSA), composed of a physician, a lawyer, and an accountant, provides regional management support to the SA program and CLAS facilities in their respective DIRESA. Non-CLAS facilities do not have such a management support team; rather, they are supervised on clinical services delivery by respective vertical health program coordinators from the DIRESA.

In summary, through the CLAS committee, community members participate in the planning, monitoring, evaluation, oversight, and accountability for implementing the local health plan; overseeing the work of health and administrative personnel under any labor regimen (public or private); hiring and firing staff under the private labor regimen; setting procurement priorities; spending oversight; health promotion planning in its jurisdiction; and others. Under CLAS law, the community is involved in actions that integrate essential functions of public health with PHC services and legal accountability mechanisms for those actions.

Previous studies comparing CLAS facilities to non-CLAS facilities have shown better performance of CLAS facilities on indicators of quality, coverage, equity, efficiency, and impact.^28^–30^,^^34^^–^^38^ For example, patient waiting times of less than 15 minutes and more than 30 minutes were found in 75% and 6% of CLAS facilities, respectively, and in 43% and 29% of non-CLAS facilities, respectively.^28^ Health service coverage was measured in a study of all 675 PHC facilities in 3 health regions (Cusco, Huánuco, and La Libertad), showing the average number of health visits per child younger than age 5 years per year as 2.32 and 2.94 in rural and urban CLAS facilities, respectively, while the averages were 1.32 and 1.73 in rural and urban non-CLAS facilities.^30^

The current study aimed to determine if good regional management practices were correlated with better performance of CLAS facilities and identify good management practices that may be linked with better PHC service performance in CLAS facilities.

## METHODS

A secondary analysis was conducted of a survey of 23 DIRESAs implemented in 2007 by the MOH of Peru. The Introduction and Discussion sections examine the relevance of reporting on the 2007 survey. The analysis aimed to assess the correlation between the use of good regional management practices and the level of service performance in CLAS comanaged facilities. Management actions were assessed through multiple-choice survey questions. More than 1 response could be marked for each question, which was classified by the authors according to the management function framework of Primary Health Care Performance Initiative^6^: leadership of the SA management team; information systems (planning, goals, monitoring); financial control; transfer of health facility management capacity and leadership skills; and supervision. A panel of experts on the SA program and CLAS classified responses as either “good practice” or “less effective practice.” We tabulated the number of good management practices reported by each DIRESA and divided that sum by the total number of possible good practices to obtain the percentage of good management practices used by each DIRESA.

A secondary analysis was conducted of a survey of 23 DIRESAs implemented in 2007 by the MOH of Peru.

As the primary outcome variable, the level of performance on delivery of PHC services was defined as the fulfillment of goals established in the local health plan of each CLAS. These goals were committed to in the comanagement agreement (contract) between each CLAS facility and its respective DIRESA. We used the inverse of the DIRESA response to the survey question, “What proportion of existing CLAS in the region have demonstrated limitations or deficiencies in meeting contracted goals?” Goals referred to the planned achievement of health coverage indicators (e.g., population-based coverage of child immunization, well child visits, and prenatal care) and health service utilization indicators (e.g., numbers of medical consultations and follow-ups, emergency visits, and dental consultations).

Pearson’s correlation coefficient was used to statistically describe the linear relationship between the percentage of good management practices used by each DIRESA and their respective average proportion of CLAS facilities with good service performance (goal achievement).

To identify specific practices associated with good performance, the DIRESAs were divided into 2 analysis groups: Group 1 with “higher-performing PHC services” and Group 2 with “lower-performing PHC services” (Table 1).

**TABLE 1.** Percentage of CLAS PHC Facilities that Completed Contracted Goals, by DIRESA and Study Group, Peru

**Group 1** ^a^	**Callao**	**Loreto**	**Tacna**	**Piura**	**Arequipa**	**Cusco**	**Total**
Facilities that met goals, no. (%)	3 (100.0)	147 (94.2)	67 (94.3)	192 (90.1)	210 (88.9)	35 (89.7)	654 (91.1)^b^
Total facilities, no.	3	156	71	213	236	39	718
**Group 2**	**Moquegua**	**Ica**	**Apurímac**	**Huánuco**	**Cajamarca**	**Ancash**	**Total**
Facilities that met goals, no. (%)	42 (73.6)	42 (70.0)	48 (67.6)	40 (35.0)	52 (20.0)	25 (20.3)	249 (37.6)
Total facilities, no.	57	60	71	114	259	123	663

Abbreviations: CLAS, Comunidad Local de Administración de Salud; DIRESA, regional health directorate; PHC, primary health care.

^a^ Completion of goals in comanagement contracts, based on local health plans.

^b^ Chi-square difference in the 2 study groups: X2=(1,N=1381)=452.82, *P*<.01.

We compared Groups 1 and 2 using chi-square to verify the differences between them in the percentage of good management practices used and in the percentage of contracted goals achieved.

To rule out external confounders that might explain the differences between the 2 study groups of DIRESAs, the groups were compared on overall regional health system capability and equity considerations by assessing the mean infant mortality rates in each of the 2 study groups during the 5-year time periods before 1996, 2007–2008, and 2014 using national demographic and health survey results.

Finally, good management practices were identified that were reported by the majority of both Groups 1 and 2 or reported more frequently in Group 1 versus Group 2.

## RESULTS

Twelve of 23 DIRESAs responded to the survey, reporting on 1,381 CLAS facilities in their regions, which comprised 64% of all 2,155 CLAS facilities nationally. On average, 65.4% of 1,381 CLAS facilities in 12 DIRESAs met the contracted goals in their respective comanagement agreement.

Thirty-two of the 52 possible management actions in the survey response options were classified as good management practice and 20 as less effective. An average of 32% of the 32 possible good practices were implemented across all DIRESAs surveyed.

The Figure shows a significant correlation between PHC services performance and the use of good regional management practices. Pearson’s correlation coefficient was r=.7266, 12 df, *P*<.01.

**FIGURE:**
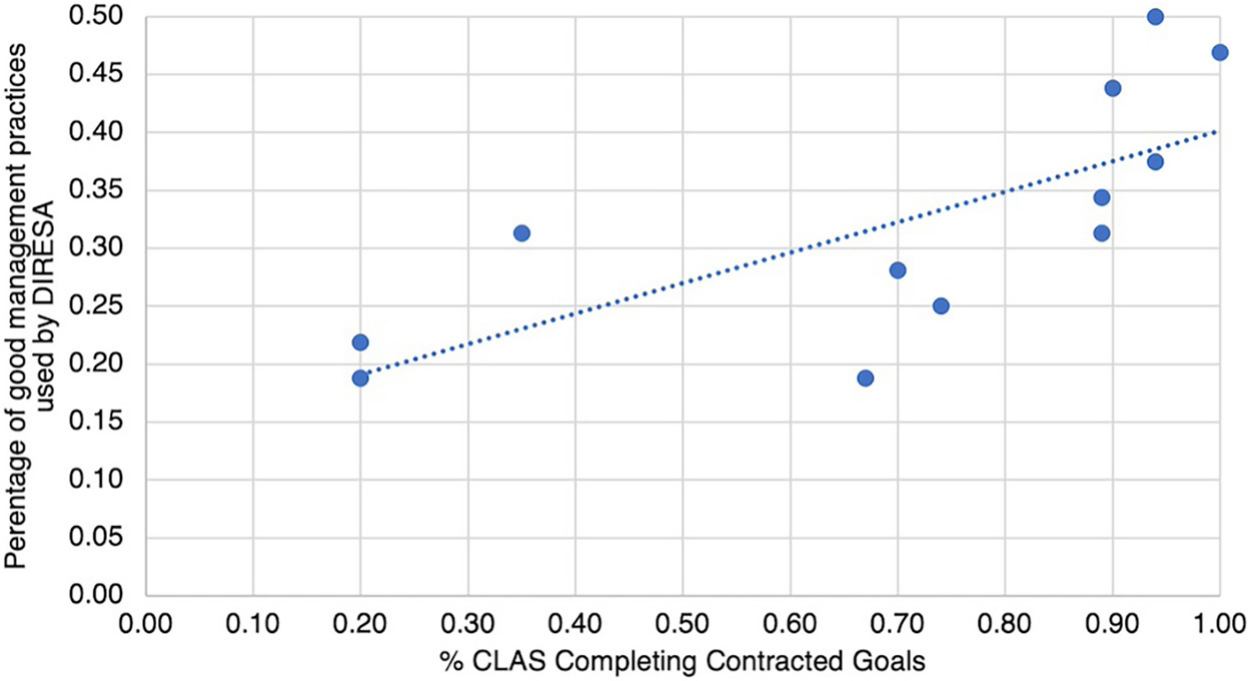
Correlation^a^ Between Percentage of CLAS Comanaged Primary Health Care Facilities That Completed Their Contracted Goals and Percentage of Good Management Practices Used by Each DIRESA Abbreviations: CLAS, Comunidad Local de Administración de Salud; DIRESA, regional health directorate. ^a^ Pearson’s correlation coefficient, r=.7266, 12 df, *P*<.01.

Dividing study DIRESAs into 2 analysis groups based on meeting performance goals, DIRESAs in Group 1 reported an average of 91.1% (range 89% to 100%) among 718 CLAS facilities that met their performance goals, while Group 2 reported an average of 37.6% (range 20% to 74%) among 663 CLAS facilities. The difference between the 2 groups is significant at *P*<.01 (Table 1).

Assessing good management practices by study group, DIRESA Group 1 used an average of 40.6% of good practices considered, significantly different from DIRESA Group 2, which used an average of 24.0% (Table 2).

**TABLE 2.** Percentage of Good Management Practices Used to Support CLAS Facilities, by DIRESA and Study Group, Peru

**Group 1** ^a^	**Callao**	**Loreto**	**Tacna**	**Piura**	**Arequipa**	**Cusco**	**Total**
Practices reported, no. (%)	15 (46.9)	16 (50.0)	12 (37.5)	14 (43.8)	11 (34.8)	10 (31.3)	78 (40.6)^c^
Practices considered, no.	32	32	32	32	32	32	192
**Group 2** ^b^	**Moquegua**	**Ica**	**Apurímac**	**Huánuco**	**Cajamarca**	**Ancash**	**Total**
Practices reported, no. (%)	8 (25.0)	9 (28.1)	6 (18.8)	10 (31.3)	7 (21.9)	6 (18.8)	46 (24.0)
Practices considered, no.	32	32	32	32	32	32	192

Abbreviations: CLAS, Comunidad Local de Administración de Salud; DIRESA, regional health directorate.

^a^ DIRESAs with a higher level of compliance with the goals contracted by CLAS.

^b^ DIRESAs with a lower level of compliance with the goals contracted by CLAS.

^c^ Chi-square difference in study groups with Yates correction: X2=(1,N=384)=11.45, *P*<.01.

DIRESA Group 1 used an average of 40.6% of good management practices considered compared to DIRESA Group 2 that used an average of 24.0%.

In this assessment of the comparability of DIRESA Groups 1 and 2 to rule out confounding characteristics of DIRESAs that could bias the correlation between the 2 main study variables, the 2 DIRESA groups were found to be similar in their mean trends in infant mortality rates over 3 5-year periods before 1996, 2007–2008, and 2014.^39^^–^^41^ These average rates per 1,000 live births were, respectively, 51, 27, and 21 in Group 1 DIRESAs and 54, 24, and 17 in Group 2 DIRESAs (Supplement 1).

### Good Management Practices in the Majority of Regions Studied

The majority of DIRESAs studied used the following 4 of 32 good management practices: (1) in 11 of 12 regions, CLAS submitted monthly financial reports with supporting documents to the DIRESA; (2) 9 of 12 DIRESAs provided technical advice to their respective CLAS associations on the formulation of their local health plan; (3) the TTSA jointly coordinated and/or conducted supervisory field visits to CLAS facilities along with supervisors of other vertical health programs; and (4) the use of a preestablished supervision guide was the case in the majority of regions.

### Good Management Practices in Regions With Better Primary Health Care Service Performance

Eleven of 32 good practices were reported more frequently in DIRESAs of Group 1 than Group 2. “More frequently” was defined as a difference of 33% or more (2 or more of 6 DIRESAs) in Group 1 reporting a certain practice compared to the number of DIRESAs reporting it in Group 2. The good management practices reported more frequently by Group 1 included 2 aspects of the TTSA formation; 1 aspect of the TTSA work planning; 1 aspect of CLAS supervision; 2 aspects of financial control; 3 aspects of technical assistance provided to CLAS; 1 aspect of human resources management; and 1 aspect of health promotion planning (Box 2).

BOX 2 Good Management Practices That Were More Frequent^a^ in Group 1 Than in Group 2

**Leadership (of the subnational management team)**

- The Shared Administration (SA) program coordinator was a regional health directorate (DIRESA) official specifically designated for that purpose.
- The coordinator of the SA program was designated by Directorial Resolution.

**Information systems (planning, goal setting, monitoring)**

- Health education and information goals were incorporated into the local health plan.

**Financial control**

- Expenses incurred by each Comunidad Local de Administración de Salud (CLAS) were monitored through periodic visits to primary health care facilities to corroborate the existence of supporting documents.
- Balance sheets of CLAS were audited by an external certified public accountant contracted by DIRESA.

**Transfer of facility management capability and leadership skills**

- Trainings for staff and CLAS community members were conducted.
- Instructional and informative documents were distributed.
- DIRESA personnel were assigned to carry out procedures for the benefit of the CLAS.

**Supervision**

- The Technical Team for the Shared Administration Program (TTSA) was clear about the actions that must be implemented for acceptable development of CLAS.
- Supervision visits to CLAS facilities were performed exclusively by TTSA members.

**Other resources provided to CLAS**

- Newly graduated health professionals were contracted short term.

^a^ More frequent defined as reported by 2 or more DIRESAs in Group 1 over the number reported in Group 2.

Detailed results of all survey questions by study group are shown in Supplement 2 (English) and Supplement 3 (Spanish).

## DISCUSSION

PHC system design should include optimal management practices to ensure quality, efficiency, and effectiveness. These are part of the structural innovations that are identified as crucial for quality services, “more likely to improve service quality substantially and at scale than are micro-level efforts.”^18^ Previous evidence has shown the positive effects of community involvement on PHC service delivery performance or quality,^26^ but few prior studies have assessed how good subnational management practices could potentiate the effect of community involvement on service improvements.

The finding that regional-level infant mortality trends are similar in DIRESA Groups 1 and 2 supports speculation that differences in the main study variables were not due to mean differences in regional capabilities, resource availability, or population characteristics between the 2 study groups.

Despite the existence of evidence of the superior impact of the CLAS comanagement model on PHC service delivery as compared to standard government PHC services (non-CLAS), health sector support for CLAS has waned over time and no longer enjoys the same level of political mandate or budgets for full implementation of the CLAS law for reasons that are important to recognize. Flagging support to CLAS may be related to the following 4 factors.

First, public sector health worker unions have threatened and conducted national strikes to get health personnel contracts changed to appointments to permanent public sector payroll, referred to as nombramiento to government service. Permanent appointments have 3 main effects on CLAS facilities, especially those in rural locations: (1) reduced PHC productivity at greater cost (lower efficiency) because the permanently appointed staff work 6 instead of 8 hours per day without proportional salary adjustment; (2) loss of PHC staff because permanently appointed staff can request transfer to urban hospitals or to publicly paid residency training, debilitating quality in public PHC services; and (3) reduced effectiveness of management through less accountability of permanently appointed staff.^42^ Medical professionals rejected the idea of being contracted by the community through CLAS facilities.^43^ They also unfairly blamed CLAS facilities for paying lower salaries, even while inadequate funds were provided for more needed staff to meet the ever-increasing demand for the better-quality services that CLAS facilities were providing. Supervision of public PHC services (mainly non-CLAS facilities) in 2018 by Controlería de la República (the national oversight agency) revealed physician productivity in number of consultations per hour to be 1.22, while the expected number is 3.72.^44^

A majority of health workers now are appointed staff with permanent public payroll status. Solutions to this issue could include providing adequate incentives (e.g., standardizing PHC worker salary levels) that would make working conditions more attractive in CLAS facilities and could constitute a superior alternative to public payroll contracts to achieve higher quality and better health outcomes for Peru’s vulnerable population that depends on public services.

A second factor that reduced support to CLAS facilities was created by the 2002 law on decentralization and 2003 law on regional governments, which were construed by subnational governments as conferring to them discretion on whether to implement national health policy. Subnational regions with all or nearly all PHC services incorporated into the SA program continue to support their CLAS facilities. Six of 25 regions have 60% to 100% of their PHC facilities incorporated into CLAS: Arequipa (100%), Tacna (100%), Madre de Dios (100%), Moquegua (91.7%), Huánuco (68.2%), and Tumbes (61.9%).^27^ Others with fewer CLAS facilities have tended to have a lower level of interest and compliance with the program. The solution to this problem would be to have a strong national mandate to implement the CLAS law and continual capacity-building of regional governments on CLAS law and regulations.

A third factor has been the introduction of a broad and well-financed plan to create Redes Integradas de Salud (integrated health networks, RIS) with cooperating networks of PHC services in 3 public health services subsystems. One is the least-financed MOH-Regional Government public system that covers 68% of the population, and the others are the Social Security subsystem (based on obligatory formal-worker payroll deductions) and the Armed Forces and Police subsystem. In the short-to-medium term, RIS creation is being applied to only the MOH-Regional Government PHC system that is managed logistically by intermediate-level health network management-budget executing units, which will continue to provide logistics management support to RIS. An RIS does not have its own logistics management unit, a mechanism for community comanagement, nor a planned system for subnational management support or oversight. The preexistence of CLAS within the jurisdiction of a new RIS has generated questions about whether CLAS can coexist with RIS, even though it is clear to many that RIS and CLAS are complementary and that, furthermore, an RIS might operate better as a CLAS.

Fourth, funding of public PHC services, including CLAS facilities, was consistently provided by the public Seguro Integral de Salud (integrated health insurance program, SIS) beginning in about 2002. However, in 2016, SIS began to instruct that funds to CLAS facilities should primarily cover variable health care costs focused on medicines, disallowing CLAS facilities to use per capita funding to hire staff. Also, SIS increasingly restricted use of funds by CLAS facilities for fixed costs, such as utilities, security, laundry and cleaning services, ambulance and building maintenance, equipment, and others. Since 2022, SIS payments directly to CLAS facilities have mostly stopped and now go to intermediate-level budget executing units, which are frequently inefficient in spending large budgets to supply PHC facilities in their jurisdiction in a timely manner for patient needs. As a result, now CLAS facilities, as well as non-CLAS facilities, frequently lack medicines, medical supplies and materials, laboratory and X-ray equipment, goods, and services.

There are advocacy efforts now to reverse these decisions. Stopping the transfer of funds to CLAS has been judged by MOH lawyers to contravene CLAS law that requires that public funds be transferred to CLAS for human resources, medicines, supplies, and other local expenses to ensure quality of care to the public.^31^

Furthermore, public health insurance reimbursements and reforms to integrate health subsectors focus primarily on clinical services and have few provisions for community health, empowerment, or social and behavior change, which now affect CLAS as well as non-CLAS facilities. The latter has long been subject to a biomedical approach to PHC provision.

Nevertheless, CLAS facilities remain an important presence in the 6 (of 25) regions in which 60% to 100% of their respective PHC services are comanaged by CLAS. That the CLAS comanagement model of PHC has survived opposition is a credit to its resiliency related to evidence-based results,^28^^–^^30^ legal stability conferred by law,^31^ and advocacy by satisfied clients who attend CLAS-run PHC services, health staff who work in CLAS facilities, and by program champions. The 4 factors previously discussed could be reversed through renewed commitment to and compliance with the CLAS law by the MOH and regional governments.

CLAS facilities remain an important presence in 6 of 25 regions in which 60% to 100% of their respective PHC services are comanaged by CLAS.

Structural aspects of community involvement and associated accountability enshrined in CLAS law provide an underlying explanation for the study findings. Empowered community that demands accountability is a key structural innovation for high-quality services that was identified in a literature review using the Lancet Global Health Commission framework on high quality health systems.^7^

### Limitations

Potential limitations to this study include that only slightly over half of DIRESAs responded to the survey. However, results do represent the situation for nearly two-thirds of all PHC facilities in the SA program comanaged by CLAS. The survey was limited in the level of detail on management actions, not all management actions were measured, nor were all factors influencing goal achievement or the relationship between management and goal achievement. The age of the survey is not a limiting factor in answering the study question because the survey reflects a time when political support for and compliance with the SA program was stronger—these being basic prerequisites for successful PHC services with community involvement—and allow an assessment of how things worked under those minimally supportive conditions.

## KEY GOOD MANAGEMENT PRACTICES

Given the significant correlation found between good subnational management practices and PHC services performance among CLAS facilities, the following 7 good management practices can and should be strengthened in community-engaged PHC systems.

**Leadership:** Clarity about responsibility is key to the proper functioning of a program.^19^ A formally recognized leader has the authority to hold team members accountable. Formalization of leadership with designation by documentary resolution is a regulatory requirement for assuming responsibilities in the Peruvian public sector. Two best practices in this study that were more frequent in Group 1 were the TTSA coordinator being expressly assigned as such and being formally designated with official documentation. Group 2 was more likely to have a TTSA coordinator who had other responsibilities, such as being the overall director of health services or coordinator of vertical health programs, with no formal designation as TTSA coordinator. Lack of formalized leadership designation may contribute to a lack of program commitment and compliance and should be included in an expanded definition of leadership in public governance.

**Information systems (planning, goals, and monitoring):** It is essential to recognize the progression of planning, implementation, and evaluation.^45^ At the time of the 2007 survey, there was no requirement that TTSAs have an annual work plan to identify and schedule management activities, which helps to explain why such plans were infrequently reported in the survey. However, TTSAs of DIRESAs in Group 1 were more often reported to be “very clear in the actions they needed to perform to ensure acceptable outcomes of the SA program” as per the survey response item. In contrast, Group 2 TTSAs made plans only periodically depending on the prioritization of needs or activities. After the survey was conducted, the central MOH formalized a requirement for TTSAs to develop a work plan.

**Health promotion planning.** Health promotion is one of the main objectives of CLAS as stated in regulations to the law.^32^ When health promotion activities are included in the local health plan to provide community health education and information and to address social determinants of health, its financing and implementation are more guaranteed through the comanagement contract.^45^^,^^46^ Specific local health promotion strategies should be designed and developed with CLAS community member participation and included in the local health plan. If health promotion is left to the discretion of each PHC service without prior inclusion in the local health plan, compliance is not assured.

**Financial control.** Financial control requires high standards and depends on an organization’s accounting and financial reporting.^47^ CLAS members and managers collaboratively manage the state’s financial resources, keep accounting books, report balances monthly to the DIRESA with supporting documents, and are lawfully responsible. Thus, transparency and financial control are more ensured. Monthly reporting by all CLAS was confirmed by 11 of 12 DIRESAs. The TTSA’s periodic visits to CLAS facilities to review supporting documents were more frequent in Group 1 than in Group 2. As for audits, DIRESA’s outsourcing to a certified public accountant provided greater objectivity and transparency. Auditing by the DIRESA’s Finance Office is also considered good practice, though it was not widely reported in the survey. It is not recommended that the audit be carried out by a certified public accountant who is part of the TTSA due to conflict of interest, nor by the DIRESA’s Internal Control Office (except in extraordinary cases).

**Financial support for the TTSA.** Neither Group 1 nor Group 2 DIRESAs in this study had a subnational program budget available to maintain the TTSA operational costs. Eleven of 12 DIRESAs received direct financial support from the central MOH office in charge of the SA program to fund TTSAs. This was a less effective management practice for its potential irregularity. When central MOH budget cuts for TTSA were instituted, some but not all DIRESAs took responsibility for these costs.

**Transfer of health facility management capability and leadership skills.** Technical support is essential for proper PHC facility functioning, whether CLAS or non-CLAS. Types of technical assistance that distinguished Group 1 from Group 2 were training provided to CLAS members and PHC staff and distribution of instructional and informative documents.

**Supervision.** Supervision should always have a prior plan with objectives, identification of operational and administrative activities and information to be supervised.^48^ Supervision of non-CLAS PHC services is oriented to the evaluation of clinical competencies and service-related information. CLAS comanaged facilities also need supervision by vertical health program managers because clinical aspects (clinical protocols, stock of drugs and supplies, health information system, and others) are the same for both CLAS and non-CLAS facilities. In contrast, TTSAs supervise and support compliance with legal, administrative, and accounting obligations of each CLAS facility, supervise the CLAS manager as executor of CLAS decisions, and monitor progress toward achievement of the local health plan. Thus, TTSA needs its own supervision guide.

The aspect of supervision that most distinguished Group 1 was that CLAS facility supervision was performed exclusively by TTSA members. Logically, supervision is most effective when supervisors are highly knowledgeable and experienced in the regulations and processes of the program to be supervised. In Group 2, supervision of CLAS by intermediate-level health network management-budget executing units was reported more frequently. Though not considered a favorable practice, this could be a good practice in the future if these units receive proper training and experience with the SA program and CLAS law and regulations.

## CONCLUSIONS

The study findings suggest that national health-related protocols, strategies, and standards may not be sufficient to achieve goals for better PHC service performance. Rather, national PHC strategies should ideally incorporate well-designed plans for operational management from the subnational level and for community-engaged PHC that is designed for accountability. Attention at the subnational level to critical aspects of PHC management (leadership of the management team, management planning, financial management, technical assistance, supervision, and local planning for health promotion and equitable access) could be fundamental for achieving sustainable improvements in health outcomes. The what and how of each management component depends on the design of the PHC-community model and must be continually reevaluated and calibrated.

Public-sector PHC services have proven deficiencies in quality, coverage, equity, efficiency, and impact, due largely to inadequate budgets but also to weak and inefficient accountability provisions, particularly in human resources management with public payroll staff, as well as extremely strict rules for procurement in public sector administration law. The PHC model of the SA program with community comanagement through CLAS is unique in the accountability embedded in its modernized contracting, procurement, and financing arrangements, allowing local decisions at the facility level that are monitored by the community. In turn, these processes are likely to be strengthened by good management from both national and subnational health offices.

Study results support recommendations for Peru to update the CLAS law to strengthen mechanisms for SA management from subnational levels; sustainably strengthen the leadership and financing of TTSA; specify TTSA roles and management instruments for planning, training, and supervision; maintain and reinstate enforcement of CLAS legislation that embeds accountability in local contracting and purchasing under private law; and resume political support for expansion of the SA program and CLAS model to improve coverage, quality, equity, and efficiency of PHC with community participation to reach goals of universal health coverage and the Sustainable Development Goals.

Study findings could help to encourage and guide other countries as they begin to redesign PHC with institutionalized community engagement that includes accountability mechanisms and intentionally align subnational management guidelines with them. It is recommended that PHC management and community engagement mechanisms be continuously evaluated to allow for effective ongoing improvements.

Further research is warranted to validate findings in similar studies with larger sample sizes, as well as studies that compare accountable community-engaged PHC with traditionally administered public PHC services.

## Notes

**Peer Reviewed**

**First Published Online:** July 29, 2024.

## References

[1] Bitton A, Ratcliffe HL, Veillard JH, et al. Primary health care as a foundation for strengthening health systems in low- and middle-income countries. J Gen Intern Med. 2017;32(5):566–571. 10.1007/s11606-016-3898-5. 27943038 PMC5400754

[2] World Health Organization (WHO). Operational Framework for Primary Health Care: Transforming Vision Into Action. WHO; 2020. Accessed July 1, 2024. https://www.who.int/publications/i/item/9789240017832

[3] World Health Organization (WHO); World Bank. Tracking Universal Health Coverage: 2023 Global Monitoring Report. WHO/World Bank; 2023. Accessed July 1, 2024. https://www.who.int/publications/i/item/9789240080379

[4] World Health Organization (WHO). UNICEF. Report of the International Conference on Primary Health Care, Alma-Ata, USSR, 6-12 September 1978 Jointly Sponsored by the World Health Organization and the United Nations Children’s Fund. WHO; 1978. Accessed July 1, 2024. https://www.who.int/publications/i/item/9241800011

[5] World Health Organization (WHO). Declaration of Astana Global Conference on Primary Health Care: Astana, Kazakhstan, 25 and 26 October 2018. WHO; 2018. Accessed July 1, 2024. https://www.who.int/publications/i/item/WHO-HIS-SDS-2018.61

[6] Primary Health Care Performance Initiative. Strong Primary health care saves lives in times of crisis and calm. Accessed July 1, 2024. https://www.improvingphc.org

[7] Kruk ME, Gage AD, Arsenault C, et al. High-quality health systems in the Sustainable Development Goals era: time for a revolution. Lancet Glob Health. 2018;6(11):e1196–e1252. 10.1016/S2214-109X(18)30386-3. 30196093 PMC7734391

[8] Demaio AR, Nielsen KK, Tersbøl BP, Kallestrup P, Meyrowitsch DW. Primary health care: a strategic framework for the prevention and control of chronic non-communicable disease. Glob Health Action. 2014;7(1):24504. 10.3402/gha.v7.24504. 25095779 PMC4122819

[9] Standing Council on Health. National Primary Health Care Strategic Framework. Commonwealth of Australia; 2013. Accessed July 1, 2024. https://extranet.who.int/countryplanningcycles/sites/default/files/country_docs/Australia/nphc_strategic_framework_final.pdf

[10] Gyawali B, Khanal P, Mishra SR, van Teijlingen E, Wolf Meyrowitsch D. Building strong primary health care to tackle the growing burden of non-communicable diseases in Nepal. Glob Health Action. 2020;13(1):1788262. 10.1080/16549716.2020.1788262. 32696724 PMC7480568

[11] Koo D, Felix K, Dankwa-Mullan I, Miller T, Waalen J. A call for action on primary care and public health integration. Am J Public Health. 2012;102(Suppl 3):S307–S309. 10.2105/AJPH.2012.300824. 22690962 PMC3478083

[12] World Health Organization (WHO). Aiming for Impact: 2024–2028 Strategy of the Alliance for Health Policy and Systems Research. WHO; 2024. Accessed July 1, 2024. https://iris.who.int/bitstream/handle/10665/376474/9789240089860-eng.pdf?sequence=1

[13] World Health Organization (WHO). Essential *Public Health Functions, Health Systems and Health Security: Developing Conceptual Clarity and a WHO Roadmap for Action*. WHO; 2018. Accessed July 1, 2024. https://apps.who.int/iris/bitstream/handle/10665/272597/9789241514088-eng.pdf

[14] Bitton A, Fifield J, Ratcliffe H, et al. Primary healthcare system performance in low-income and middle-income countries: a scoping review of the evidence from 2010 to 2017. BMJ Glob Health. 2019;4(Suppl 8):e001551. 10.1136/bmjgh-2019-001551. 31478028 PMC6703296

[15] Muñoz F, López-Acuña D, Halverson P, et al. Las funciones esenciales de la salud pública: un tema emergente en las reformas del sector de la salud. Rev Panam Salud Publica Pan Am J Public Health. 2000;8(1/2):126–134. Accessed July 1, 2024. https://www.scielosp.org/article/ssm/content/raw/?resource_ssm_path=/media/assets/rpsp/v8n1-2/3012.pdf 10.1590/s1020-49892000000700017 11026783

[16] Panamerican Health Organization (PAHO). Las Funciones Esenciales de la Salud Pública en las Américas. Una Renovación Para el Siglo XXI. Marco Conceptual y Descripción. PAHO; 2020. 10.37774/9789275322642

[17] Berman P, Bitrán R. Health Systems Analysis for Better Health System Strengthening. World Bank; 2011. Accessed July 1, 2024. https://www.jstor.org/stable/resrep26277

[18] Kruk ME, Lewis TP, Arsenault C, et al. Improving health and social systems for all children in LMICs: structural innovations to deliver high-quality services. Lancet. 2022;399(10337):1830–1844. 10.1016/S0140-6736(21)02532-0. 35489361 PMC9077444

[19] Puertas EB, Sotelo JM, Ramos G. [Leadership and strategic management in health systems based on primary health care]. Rev Panam Salud Publica. 2020;44:e124. 10.26633/RPSP.2020.124. 33165409 PMC7603369

[20] Woolcock M. Enhancing public health outcomes in developing countries: from good policies and best practices to better implementation. Scand J Public Health. 2018;46(22_suppl):10–18. 10.1177/1403494818765690. 29862909

[21] Ministerio de Salud del Perú (MSP). Ministerio de Salud, Dirección General de Salud de Las Personas. Plan Nacional de Fortalecimiento Del Primer Nivel de Atención 2011-2021. MSP; 2011. Accessed July 1, 2024. http://bvs.minsa.gob.pe/local/MINSA/1620.pdf

[22] Ministerio de Salud del Perú (MSP). Documento Técnico: Modelo de Cuidado Integral de Salud por Curso de Vida Para la Persona, Familia y Comunidad (MCI). MSP; 2021. Accessed July 1, 2024. https://bvs.minsa.gob.pe/local/fi-admin/rm-220-2021-minsa.pdf

[23] Ley N^o^ 30885, Ley que establece la conformación y el funcionamiento de las Redes Integradas de Salud - RIS. Ministerio de Salud del Perú. Accessed July 1, 2024. https://busquedas.elperuano.pe/dispositivo/NL/1724734-2

[24] Decreto Supremo No 019-2020-SA, que aprueba el Reglamento de la Ley N° 30885, Ley que establece la conformación y el funcionamiento de las Redes Integradas de Salud - RIS. Ministerio de Salud del Perú. Accessed July 1, 2024. https://busquedas.elperuano.pe/dispositivo/NL/1866899-12

[25] Espinoza-Portillo E, Gil-Quevedo W, Agurto-Távara E. Principales problemas en la gestión de establecimientos de salud en el Perú. Rev Cuba Salud Pública. 2020;46(4). Accessed July 1, 2024. http://scielo.sld.cu/scielo.php?script=sci_arttext&pid=S0864-34662020000400012#B3

[26] Dodd R, Palagyi A, Jan S, et al. Organisation of primary health care systems in low- and middle-income countries: review of evidence on what works and why in the Asia-Pacific region. BMJ Glob Health. 2019;4(Suppl 8):e001487. 10.1136/bmjgh-2019-001487. 31478026 PMC6703302

[27] Cruz E, et al. Sistematizacion de La Situación Del Modelo de Cogestión. DIPOS-Dirección General de de Aseguramiento y Intercambio Prestacional, Ministerio de Salud; 2018.

[28] Cortez R. Equidad y Calidad de Los Servicios de Salud: El Caso de Los CLAS. Universidad de Lima; 1998. Accessed July 1, 2024. https://faculty.up.edu.pe/es/publications/equidad-y-calidad-de-los-servicios-de-salud-el-caso-de-los-clas

[29] Altobelli LC. Pancorvo J. El Programa de Administración Compartida y los Comités Locales de Administración de Salud: Estudio de Caso del Peru. World Bank; 2000.

[30] Altobelli LC. Sovero A. Cost-Efficiency of CLAS. Future Generations, DFID; 2002.

[31] Ley que Establece la Cogestión y la Participación Ciudadana en los Establecimientos de Salud del Primer Nivel de Atención del Ministerio de Salud y de las Regiones. Ley No 29124. Diario El Peruano. 30 octubre 2007. Congreso de la Republica.; 2007.

[32] Decreto Supremo que aprueban el Reglamento de la Ley N° 29124 que establece la cogestión y participación ciudadana en los establecimientos de salud del primer nivel de atención del Ministerio de Salud y de las Regiones. Decreto Supremo No 017-2008-SA. Diario El Peruano. Congreso de la Republica.; 2008. Accessed July 1, 2024.

[33] Ley de Bases de la Descentralización, Ley 27783 (Internet). Diario El Peruano. 20 julio 2002. Congreso de la Republica.; 2002. Accessed 1, 2024. https://www.leyes.congreso.gob.pe/Documentos/Leyes/27783.pdf

[34] Montes Gómez B, Martel Carranza CP, Torero Solano de Martel NZ. Cogestión en los servicios de salud - Huánuco, Peru. Revista peruana de ciencias de la salud. 2020;2(3):70–76. 10.37711/rpcs.2020.2.3.196

[35] Altobelli LC. Comparative analysis of primary health care facilities with participation of civil society in Venezuela and Peru. In: Interamerican Development Bank; 1998. Accessed July 1, 2024. https://www.researchgate.net/publication/351885856_COMPARATIVE_ANALYSIS_OF_PRIMARY_HEALTH_CARE_FACILITIES_WITH_PARTICIPATION_OF_CIVIL_SOCIETY_IN_VENEZUELA_Y_PERU_Social_Programs_Poverty_and_Citizen_Participation

[36] Vicuña M, Ampuero S, Murillo JP. Analisis de la demanda efectiva y su relacion con el modelo de gestion en los establecimientos del primer nivel de atencion. Estudio de evaluación del Programa de Administración Compartida. Ministerio de Salud-PAAG-SBPT-AC; 1999.

[37] Altobelli LC. Acosta-Saal C. Local Health Administration Committees (CLAS): opportunity and empowerment for equity in health in Peru. In: Blas E, Sommerfeld J, Kurup AS. *Social Determinants Approaches to Public Health: From Concept to Practice.* World Health Organization; 2011. Accessed July 1, 2024. https://apps.who.int/iris/handle/10665/44492

[38] Altobelli LC. Aid Effectiveness in Primary Healthcare in Peru. In: E. Beracochea *(Ed.)* Improving Aid Effectiveness in Global Health. Springer Global Publishing; 2015. Accessed July 1 2024. https://www.researchgate.net/publication/266793303_Aid_Effectiveness_in_Peru_How_a_bottom-up_health_reform_model_strengthens_organizational_and_management_structures_to_effectively_utilize_national_and_donor_resources

[39] Instituto Nacional de Estadística e Informática. Demographic and Health Survey. Encuesta Demografica y de Salud Familiar 1996. Instituto Nacional de Estadística e Informática; 1997. Accessed July 1, 2024. https://dhsprogram.com/pubs/pdf/FR87/FR87.pdf

[40] Instituto Nacional de Estadística e Informática. Demographic and Health Survey. Encuesta Demografica y de Salud Familiar 2007-2008. Instituto Nacional de Estadística e Informática; 2007. Accessed July 1, 2024. https://dhsprogram.com/pubs/pdf/fr234/fr234.pdf

[41] Instituto Nacional de Estadística e Informática. Demographic and Health Survey. ENDES 2014. Instituto Nacional de Estadística e Informática; 2014. Accessed July 1, 2024. https://dhsprogram.com/pubs/pdf/FR310/FR310.pdf

[42] Lenz R, Alvarado B. Pro-poor policies in the public health sector in Peru. In: D. Cotlear *(Ed.):* A New Social Contract for Peru. How to Achieve a Country with More Education, Health, and Solidarity? World Bank.; 2006.

[43] Ugarte O. Personal communication. Presented at: Miercoles de Salud Publica: Hablemos de la Solucionática; January 24, 2024; Universidad Peruana Cayetano Heredia.

[44] Controleria de la Republica. Physician Productivity in Public Sector Services. Controleria de la Republica; 2018.

[45] Terry PE. Health promotion planning and an interview with Dr. Lawrence Green. Am J Health Promot. 2021;35(6):760–765. 10.1177/08901171211022560. 34120469

[46] Kim RW, Nahar VK. A guide for understanding health education and promotion programs. Health Promot Pract. 2018;19(2):167–169. 10.1177/1524839917741487. 29130751

[47] Drakic-Grgur M. Financial management. Stud Health Technol Inform. 2020;274:68–81. 10.3233/SHTI200667. 32990666

[48] Bosch-Capblanch X, Garner P. Primary health care supervision in developing countries. Trop Med Int Health. 2008;13(3):369–383. 10.1111/j.1365-3156.2008.02012.x. 18397400

